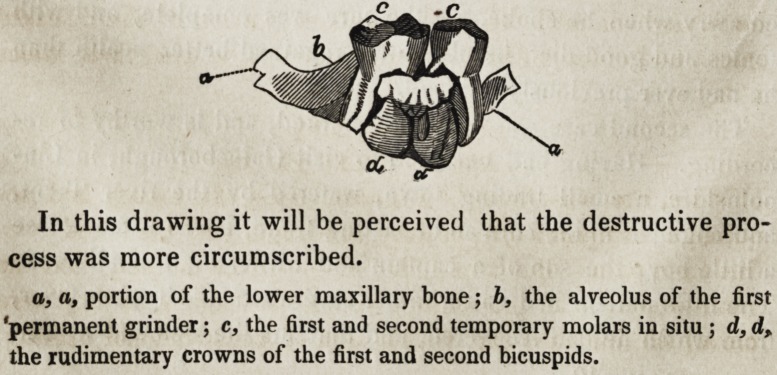# Cases of Cancrum Oris

**Published:** 1851-10

**Authors:** J. L. Levison

**Affiliations:** Brighton, Eng.


### ARTICLE X.

*Cases of Cancrum Oris.* By J. L. Levison, of Brighton, Eng.

Mr. Editor—As a proof of the esteem I entertain for the American dentists, I willingly (at your request) send an article for your Journal, promising other communications should you desire them. In the Lancet of last Saturday, (June the 7th, 1851,) there is an interesting case of cancrum oris reported, as under the care of Dr. Burrows of St. Bartholomew's Hospital, the patient being thirteen years old. In the introductory remarks it is stated, that the disease is attributed to debilitating causes, and that it often supervenes on exanthematous diseases, &c. And as the subject is one of interest to the practical dentist, I will select a few cases from my note book, as furnishing *data* of some of the predisposing causes for the development of this affection, the most malignant instances in my own practice, I observed during my residence at Hull, which was almost endemical among the poor, in the worst localities of that town. It should be premised, that Hull is built on an alluvial deposit of mud thrown up from the river Humber, that it is situated very low, having also canals intersecting all parts of the town ; and besides, there is the river Hull on one of its sides, and the river Humber on the other. It is, therefore, generally damp, which singly often causes great debility, &c., that dyspepsia is an endemical disorder of the place. Besides which, it is always more or less subject to low nervous and typhoid fevers, and suffered a great mortality during both visitations of the Asiatic cholera. I was asked, on one occasion, by a surgeon, to look at the mouth of a boy about eight years old, who had had a severe attack of typhus fever. The poor little fellow was naturally lymphatic, and of a strumous habit; that though the fever had left him, he was represented to be in a dangerous state, from a grangrenous form of inflammation of the mouth, and from the swollen state of the face on one side, it was conjectured, that there existed a deep seated abscess, and that it extended from the center of the mouth to the coronoid process. When I first saw him, he appeared to be sinking from constant pain and inability to eat. The room was sickening to enter, from the fetid atmosphere, and on approaching the little sufferer, his breath caused us to experience a most painful nausea. However, I persisted on examining his mouth, and instead of an abscess, I observed the whole half of the lower jaw, from the symphysis of the maxillary bone to the coronoid process, completely denuded of the gum, and presenting well marked data to determine that the exposed bone was in a state of necrosis ; and that the local inflammation and the extensive formation of pus, arose from an effort of nature, to throw off the dead substance. We differed, however, as to the stage of the process, as an opinion was offered, that it might be months before the separation could take place. But, as I did not rest satisfied with a mere ocular inspection, I felt the dead bone with my finger and thumb, and found that I could move the whole with the teeth in it. So, to give myself less personal annoyance, and to expedite the process, I ordered the following lotion, aqua rosæ ℥ viij and xii drops of nitric acid, to be frequently injected with a syringe in the diseased part of the mouth. The next day I found the mouth much cleaner, and with comparatively little fetor, and perceiving that the diseased bone was only connected at its extreme posterior edge by a portion of gum resembling cartilage, I took a small curved instrument, (like a flattened tenaculum,) and easily separated the whole portion of jaw, a drawing of which is annexed.

*a, a,* half the lower jaw, from the symphysis ; *b,* socket of the first permanent molar, which has fallen out, and has been lost ; *c,* second temporary molar ; *d,* alveolus of the first temporary molar ; *e,* alveolus of the temporary cuspidatus ; *f, f,* the alveoli of the permanent lateral and central incisors.

The boy rapidly recovered, and the loss of the bone was replaced by a hard cartilaginous substance, so that the face externally did not show any deformity; though, in speaking, the expression was not natural, as the buccinator muscle had become attached to the newly formed substance, so that the mouth looked awry when he spoke, yet the cure was complete, and with tonics and good diet, he ultimately regained better health than he had ever previously enjoyed.

The second case was also well marked, and is worthy of recording. Having had occasion to visit Gainsborough, in Lincolnshire, a small trading town, watered by the river Trent, and engaged in the corn and coasting trade, I was asked to see a little boy, the son of a captain and owner of a small craft. The little patient had had a rather severe attack of typhus fever, from which he had recovered, and that his life was endangered by what appeared to be a case of cancrum-oris. The whole mouth had a gangrenous appearance. It was much swollen, and caused constant pain and inconvenience. I should mention that the town of Gainsborough, like Hull, is a damp, low place, and surrounded by a flat marshy country, having the river Trent on one of its sides, with long, narrow and confined streets, thickly inhabited, particularly in the vicinity of the river. The latter part mentioned, is very sickly, and often visited by contagious fevers and epidemic affections; during the time of the Asiatic cholera, the disease raged in this locality, and left many proofs of its ruthfulness. The subject, however, of this brief sketch, occupied a house in a more healthy part of the town, and from the circumstances of his parents, he had not suffered from any deficiency of diet, which is one of the predisposing causes of this form of the disease of the mouth.

He was a nice lad, about 8 or 9 years old, strongly made, and of a nervo-sanguinous temperament—he had a good and well formed head, and indicated great natural intelligence, and showed its influence by the fortitude with which he bore his constant suffering. On looking into his mouth, I perceived a portion of necrosed bone, with the teeth in it, being a portion of the lower jaw. It seemed loose and detached, and this was the result of his natural vigor of constitution and the active inflammation he had endured, which had effected the process of separation, and there was little trouble in removing the offending and extraneous body.

In this drawing it will be perceived that the destructive process was more circumscribed.

*a, a,* portion of the lower maxillary bone; *b,* the alveolus of the first permanent grinder; *c,* the first and second temporary molars in situ; *d, d,* the rudimentary crowns of the first and second bicuspids.

M—— E——, a girl about seven years old, residing in this town, was brought to me for my advice. She is the daughter of a jobbing shoemaker, or rather mender, who looks of a sickly, dirty yellow color, and her mother is a pale, weak looking, nervous person, who assisted in obtaining the small wants of her family by mangling: and as there happens to be about this case, incidentally, a great amount of interest, it seems not only worthy, but very important to be thus particularized. M—— E—— was herself, a short, pale-faced looking child, with a large head, having had, in all probability, during her infancy, hydrocephalus internus, but which had been cured. She could not stand, much less attempt to walk. The mother was therefore, obliged to carry her in her arms when she sought my advice. On looking into her mouth, I decided that the affection was much like cancrum oris. The gums, for example, had a gangrenous aspect, being livid and gorged with a dark purplish red colored blood, and the fetor extreme. Besides which, the gums had grown in such a preternatural manner, as to form a complete covering for every tooth; nay, hanging over them pendulous, enabling these flaps to be lifted up, sufficiently high to observe the dirty, yellow looking teeth they otherwise concealed. On the right side of both upper and lower jaws, there were abscesses, arising from a number of loose teeth, which were carious, and which presented sharp and angular edges to keep up a constant irritation—whilst the pus as it oozed out mixed with the saliva, which on being swallowed, kept up a constant nausea. I, therefore, removed all the loose teeth, and excised the superabundant portion of the diseased gums, and ordered the following lotion to be used three times a day.

Aqua rosæ, ℥ vij.

Argent. nitrat. gtt. xij.

When she was brought a second time, the mouth had acquired a comparatively healthy condition, and the child was free from pain. But I told Mrs. E——, (the mother,) that the gums would, in all probability, become bad again, as they were merely the outward and visible sign of constitutional debility.

So I gave her a note to a young German physician, requesting him to give the bearer his gratuitous services. I could not help smiling at the child's protest, "Pray sir, don't send me to a doctor, as I have taken a wagon load of physic." The sickly-faced mother also smiled at the energy of her afflicted girl, and said, "She has, indeed, had many doctors." But she could not stand, nor had she been able to make the attempt for some time past. My German friend declined the case, as he despaired of curing her after so many had failed. The poor woman returned to tell, with a countenance expressive of despair. Prompted by humanity, I examined the spine, and found the second and third lumbar vertebræ forced outwards, resembling a bony excrescence. As I felt assured the child had worms, I urged her to try a simple German remedy, namely, to take a modern sized silver tea-spoonful of common salt, (the chloride of sodium,) in a tumbler of tepid water, an hour before rising in the morning. My advice was given on Friday, and on the following Monday, Mrs. E—— came to tell me, with tears rolling down her cheeks, that her poor invalid had voided three pints of worms. I recommended to continue the same treatment for some weeks, and to place her child on a hard mattress, with a padding under her, to press on the protruded bones, and soon the latter assumed their proper place, and I had the pleasure of seeing the little dwarfish, intelligent child, walk down to my house to thank me.

In this case we have indubitable evidence of the intimate connection of the teeth with the mucous tissues: as the intestinal irritation had induced extensive irritation in the buccal cavity, involving the organs of the mouth in general, and of the gums in particular; hence, teaching a most important lesson, viz. that in forming a diagnosis, we should endeaver to obtain from the patient something of his or her previous history. For, otherwise, grave errors may be committed in practice. If, for instance, a medical man should always pronounce every case as cancrum oris where the gums were enlarged and gangrenous, and the alveoli exposed, to be the sequala of exanthematous diseases, he would in all such cases (as the one now recorded)

fail to cure. Where, however, the cause is local, as in the two previously described cases, the cures were perfect, and the previous disturbance to the health (induced by the inflammation of the mouth) presented all the testimony of cause and effect. But this would not have been the case with M—— E——. The relief afforded by the removal of the carious teeth and morbidly increased gums, would only have been temporary in its consequences : for whilst the lower bowels were loaded with worms, the mere local habitation, they would have been highly irritative to the mucous surfaces, and this irritation would have again affected the mouth, it being a mere extension or continuation of its surface, and the poor child would have most likely had a repetition of the symptoms for which I was first consulted. Lastly, her stunted growth of body, evidently too short for her large head, and the weakness of the lower extremities, seem to warrant the conclusion, *a priori*, that the functions of the spinal nerves were greatly interfered with, and all the chylopoietic viscera (particularly the liver) more or less imperfectly performing their functions from hereditary influences.

## Figures and Tables

**Figure f1:**
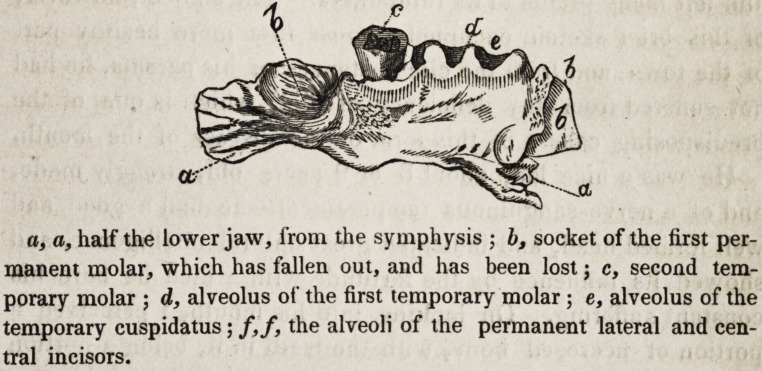


**Figure f2:**